# Cover crop inclusion and residue retention improves soybean production and physiology in drought conditions

**DOI:** 10.1016/j.heliyon.2024.e29838

**Published:** 2024-04-20

**Authors:** Craig W. Whippo, Nicanor Z. Saliendra, Mark A. Liebig

**Affiliations:** USDA-ARS, Northern Great Plains Research Laboratory, P.O. Box 459, Mandan, ND, 58554, USA

**Keywords:** Big-leaf, Drought stress, Soybean, Management practices, Northern great plains

## Abstract

Soybean (*Glycine* max (L.) Merr.) planting has increased in central and western North Dakota despite frequent drought occurrences that limit productivity. Soybean plants need high photosynthetic and transpiration rates to be productive, but they also need high water use efficiency when water is limited. Crop residues and cover crops in crop rotations may improve soybean drought tolerance in northern Great Plains. We aimed to examine how a management practice that included cover crops and residue retention impacts agronomic, ecosystem water and carbon dioxide flux, and canopy-scale physiological attributes of soybeans in the northern Great Plains under drought conditions. The experiment consisted of two soybean fields over two years with business-*as*-usual (no-cover crops and spring wheat residue removal) and aspirational management (cover crops and spring wheat residue retention) during a drought year. We compared yield; aboveground biomass; green chromatic coordinates, and CO_2_ and H_2_O fluxes from eddy covariance, Phenocam images, and ancillary micrometeorological measurements. These measurements were used to derive ecosystem-scale physical, and physiological attributes with the ‘big leaf’ framework to diagnose underlying processes. Soybean yields were 29 % higher under drought conditions in the field managed in a system that included cover crops and residue retention. This yield increase was associated with a 5 day increase in the green-chromatic-coordinate defined maturity phenophase, increasing agronomic and intrinsic water use efficiency by 27 % and 33 %, respectively, increasing water uptake, and increasing the rubisco-limited photosynthetic capacity (V_cmax25_) by 42 %. The inclusion of cover crops and residue retention into a cropping system improved soybean productivity because of differences in water use, phenology timing, and photosynthetic capacity. These results suggest that farmers can improve soybean productivity and yield stability by incorporating cover crops and residue retention into their management suite because these practices to facilitate more aggressive water uptake.

## Highlights

• Soybean fields differing in cover crop use and residue management were compared.
• A big-leaf framework was used to evaluate ecophysiological attributes.
• Cover crops and residue retention improved soybean yield, water-use, and photosynthetic potential during drought.
• Farmers can increase soybean production by using cover crops and retaining residue.

## Abbreviations

AGB: aboveground biomass
AGDD: accumulated growing degree days
ASP: aspirational
BAU: business as usual
C_surf_: surface CO_2_
DOY: day of year
DT: day time
EC: eddy covariance
EOS: end of season
ER: ecosystem respiration
ET: evapotranspiration
G_1,uso_: stomatal slope
G_ah_: aerodynamic conductance to heat transfer
GCC: green chromatic coordinate
GEP: gross ecosystem productivity
GPP: gross primary productivity
GY: grain yield
HI: harvest index
J_max_: maximum electron transport rate
LAI: leaf area index
LE: latent heat flux
NEE: net ecosystem exchange
NEP: net ecosystem production
NGP: northern Great Plains
NT: night time
PPFD: photosynthetic flux density
PCPN: precipitation
R1: beginning bloom
R2: full flowering
SE: standard error
SOG: start of green up
SOM: start of maturity
SOS: start of senescence
SWC: soil water content
T: temperature
V4: unifoliolate and 4 trifoliolate leaves developed
V9: unifoliolate and 9 trifoliolate leaves developed
VC: cotyledon stage
V_cmax35_: maximum carboxylation rate at 25 °C
VE: emergence stage
VPD: vapor pressure deficit
WUE: water use efficiency

## 1 Introduction

For most of the 20th century, cropping systems in the northern Great Plains (NGP) followed a sequence of spring wheat (*Triticum aestivum* L) and summer fallow. This cropping system was developed for the short growing season and semi-arid climate of the NGP. Since the 1980s, soybean (*Glycine* max (L.) Merr.) production has been expanding westward in North Dakota due to a combination of climate change, the release of short-season and herbicide-resistant varieties, and the adoption of no-till management practices [1]. Over this time, soybean seed yield under near-normal precipitation conditions has increased nearly four-fold [2,3].

Drought during the growing season limits soybean production due to restricted water availability [2]. The average precipitation accumulation during the growing season (May 15 to September 15) from 1921 to 2021 is 250 mm based on data obtained from the National Oceanic and Atmospheric Administration weather station located at the Mandan Experiment Station (GHCND:USC00325479) [4]. The 2020 drought continued into 2021 resulting in the sixth driest year (120 mm) and the 33rd warmest year (6.8 °C) on record between 1921 and 2021. Drought frequency and duration are expected to increase in the NGP [5]. Therefore, expansion of soybean into southwestern North Dakota comes with significant production risks requiring innovative management systems that improve soybean performance under drought conditions.

To cope with drought conditions, soybean plants (and other grain legumes) can conserve soil water until grain fill is complete, or they can withdraw water from the soil and take advantage of transitory increases in soil moisture due to precipitation events [6]. Since nitrogen fixation is energetically expensive, soybean plants need to have high photosynthetic and transpiration rates to be productive, but they also need high water-use efficiency when water is limited [7,8]. Plants can conserve and acquire water through several strategies at the individual trait level, but each of these strategies has corresponding tradeoffs. These strategies include regulating traits that control plant water status such as root architecture, sensitivity of stomatal conductance (g_s_) to vapor pressure deficit (VPD), hydraulic conductivity, and osmotic adjustment. The water status of a plant is controlled by traits that affect stomatal conductance [6]. The photosynthetic rate is driven by intercepted light but determined by g_s_ and photosynthetic capacity, which can be measured by maximum rates of carboxylation and electron transport [9].

Increasing residue retention can decrease soil evaporation and trap more snow during the winter, thus potentially increasing availability during the growing season [[10], [11], [12]]. Therefore, residue retention can improve soybean yield [13] and soil properties [14]. However, cool temperatures slow residue decomposition and inhibit nitrogen mineralization [15], which may contribute to lower soybean yields in humid northern climates [16,17]. However, in semi-arid northern climates such as the NGP the effects of residue retention on soybean requires more characterization.

Including cover crops in a crop rotation may enhance soybean production in the NGP. Rye (*Secale cereale*) incorporated as a cover crop into a central Pennsylvania soybean-corn-wheat rotation improved arbuscular mycorrhizal fungi colonization, weed suppression, and increase the presence of beneficial insect [18]. Under drought conditions in Maryland, soybean yield was improved by root channels created by the decomposition of the previous radish (*Raphanus sativus*)-rye cover crop and soil water conservation due to the rye mulch [19]. The benefits of incorporating cover crops into soybean rotations may be more limited in the NGP due to the short growing season for cover crops, nitrogen immobilization, and potential for increased water stress [20,21].

To address questions regarding how to sustainably intensify and manage agriculture, the USDA Long-Term Agroecosystem Research (LTAR) Network began a ‘common experiment’ across 18 sites throughout the country [22]. Each of these sites is conducting experiments comparing business-as-usual management practices with alternative management approaches that hold promise for improving the long-term management of natural resources in agricultural production. Thirteen of these sites have eddy covariance towers with ancillary micrometeorological measurements and Phenocams [23] to compare carbon dioxide and water fluxes over long periods of time with high temporal resolution in croplands [24]. These measurements allow for site-level and network level comparisons between contrasting management practices. Carbon and water flux data from the NGP LTAR fields have two of these eddy covariance towers have been previously reported [3,24,25], including flux measurements from 2018, when soybean was planted in these fields using the business-as-usual management practices [3]. The NGP LTAR fields have the lowest mean annual temperature (5.1 °C), mean annual precipitation (496 mm), average annual gross primary production (GPP, 601 g m^2^), and average annual ecosystem respiration (Reco, 595 g m^−2^) of the six LTAR locations growing soybean [24]. Thus, this site offers novel information about the carbon and water fluxes of soybean at the geographic fringe of soybean production.

Detailed evaluations of underlying crop physiological processes are typically time sensitive and time intensive. Data with high temporal resolution from eddy covariance can provide a field-level view of energy, carbon dioxide, and water fluxes in response to environmental factors for the cropping system. Other ecophysiological attributes can be estimated from eddy covariance data using a ‘big leaf’ framework to provide comparable mechanistic insights across eddy covariance sites with minimum ancillary data requirements as long as the interpretation is limited to bulk ecosystem properties [26]. The ‘big-leaf” framework assumes that all fluxes originate from a single plane, and derived values are treated as the average of that surface at the tower footprint. Lastly, the ‘big-leaf’ framework can be augmented by PhenoCam imaging to identify the time period associated with maximum LAI or peak biomass.

We used eddy covariance data from two LTAR fields in the NGP to compare how business-as-usual as commonly practiced by local producers (without cover crops and residue removal) and aspirational management (with cover crops and spring wheat residue retention) affect the agronomic, phenological, and ecophysiological attributes of soybean during an unprecedented drought in 2021. We hypothesized that soybean grown in the aspirational fieldwould have better performance characteristics under drought conditions than the business-*as*-usual field. To test this hypothesis, we examined the divergence between business-*as*-usual and aspirational fields by measuring agronomic and environmental attributes and deriving ecophysiological properties using the ‘big leaf’ framework.

## 2 Methods

### 2.1 Study location and management

The research was conducted at the Area IV Soil Conservation Districts Cooperative Research Farm, which is situated 5 km southwest of Mandan, North Dakota, USA (46.77, −100.95; 535 m asl). The investigation was conducted in two fields, labeled as H5 and I2, with soils classified as Temvik-Wilton silt loams. Fields H5 and I2 were 19.4 and 22.3 ha in size, respectively, and were separated by 2.5 km. Soil conditions in the surface 30 cm were non-saline (<2 dS m^−1^), strongly to slightly acidic (5.33–6.51; 1:1 soil pH), moderately fertile (2.7–4.9 % soil organic matter), with medium to moderately-fine soil textures (Table S1). The terrain at field H5 was mostly flat (0–1% slope). The terrain at field I2 was characterized by gentle undulations with a slope ranging from 0 to 3 %. The climate at the fields is classified as semiarid continental [27].

From 1984 through 2015 fields were managed as rainfed cropping systems as reviewed by Liebig et al. [3]. Between 2016 and 2018, both fields were cropped with a spring wheat – corn – soybean rotation under no-tillage management. Crop selection, sequencing, and associated practices were reflective of rainfed management systems in the region and were designated as ‘Business as Usual’ (BAU).

In 2019, fields were assigned different treatments, with field H5 continuing under BAU management while field I2 transitioned to aspirational (ASP) management. The ASP treatment maintained the same three-year crop rotation, but included a three-way cover crop mix (pea [*Pisum sativum* L.], winter wheat [*Triticum aestivum* L.], and radish) planted after spring wheat harvest with a JD750 no-till drill (Deere & Company, Moline, IL), while a four-way cover crop mix (rye, triticale [ × Triticosecale Wittmack], cowpea [*Vigna unguiculata* (L.) Walp. ], purple top turnip [*Brassica campestris* L.]) was interseeded in corn at V4-stage with an InterSeeder Planter (InterSeeder Technologies LLC, Woodward, PA) [28]. The biomass of the cover crop in the ASP field was left unharvested. Cover crops that survived winter were permitted to grow in the spring but were terminated with herbicide immediately prior to field activities for the subsequent crop. Residue management between BAU and ASP fields differed, as spring wheat grain from the ASP field was harvested with a stripper header resulting in standing residue 0.3–0.4 m in height, while wheat grain from the BAU field was harvested with a flex head set at 0.15 m from the soil surface. Crop varieties, cover crop selection, seeding dates and rates, fertilizer and pesticide applications, and grain harvest followed methods commonly used by farmers in the region (Table 1).

**Table 1:** Field operations over a four-year period including soybean-spring wheat-corn-soybean (2018–2021) at the LTAR Northern Plains Croplands Common Experiment research site, Mandan, ND, USA.

Operation	2018 (soybean)		2019 (spring wheat)		2020 (corn)		2021 (soybean)	
	BAU (H5)	BAU (I2)	BAU (H5)	ASP (I2)	BAU (H5)	ASP (I2)	BAU (H5)	ASP (I2)
Cropplanting								
Variety	Mycogen 5B024		ND VitPro		Mycogen 2J238R2		Croplan CP0329E	
Date (mm/dd)	05/30	05/31	05/17	05/16	05/20-21	05/22-25	05/27	06/01
Rate (seeds ha^−1^)	543,400		3.2 million		59,280		432,250	
Fertilizer								
Date (mm/dd)	None	None	04/15; 05/17	04/15; 05/16	04/17	04/17	4/30	None
Nutrient/Rate (kg ha^−1^)			N/65; P/17	N/46; P/17	N/101	N/101	N/33	
Cover crop planting								
Date (mm/dd)	None	None	None	09/05-11	None	06/24-26	None	None
Crop/Rate (seeds ha^−1^)			–	Vine pea/124,000 Winter wheat/478,000 Oilseed radish/31,000	–	Winter rye/747,000 Spring triticale/103,000 Cowpea/218,000 Purple top turnip/882,000	–	–
Herbicide application								
Date (mm/dd)	05/22; 06/27	05/22; 06/27	06/17	06/17	05/16; 06/23	05/16; 06/23	06/28	05/06; 06/28
Rate (kg ha^−1^, a.i.)	2.0 glyphosate, 0.2 sulfent, 0.03 carfent; 2.0 glyphosate, 0.07 quizalofop-P-ethyl	2.0 glyphosate, 0.2 sulfent, 0.03 carfent; 2.0 glyphosate, 0.07 quizalofop-P-ethyl	0.11, clopyralid; 0.11, fluroxypyr; 0.26, 2,4-D	0.11, clopyralid; 0.11, fluroxypyr; 0.26, 2,4-D; 0.01, halauxifen; 0.01 florasulam	1.42, glyphosate (applied twice); 0.07, dicamba; 0.43 2,4-D	1.42, glyphosate (applied twice); 0.07, dicamba; 0.43 2,4-D	1.42 glyphosate	1.42 glyphosate; 0.14 dicamba; 0.40 2,4-D
Fungicide application								
Date (mm/dd)			06/17	06/17				
Rate (kg ha^−1^, a.i.)			0.13, propiconazole	0.13, propiconazole				
Grain harvest								
Date (mm/dd)	10/17	10/16	09/17	08/28	10/05	10/08-16	10/29	11/04

### 2.2 Micrometeorological measurements

Continuous measurements of eddy covariance, meteorological, and soil moisture were undertaken in both 2018 and 2021 as previously described [3,29]. Briefly, a tripod held an n open-path gas analyzer (model LI-7500A; Li-Cor Inc., Lincoln, NE) and a sonic anemometer (model CSAT3; Campbell Scientific Inc., Logan, UT) 2.8 m above the ground. Sonic anemometer and gas analyzer time series data were recorded at 10 Hz.

### 2.3 Processing of eddy covariance data

Details on the processing of eddy covariance have been described elsewhere [3,29]. The online tool, REddyProcWeb, was used in gap-filling and flux-partitioning of half-hourly data (https://www.bgc-jena.mpg.de/REddyProc/ui/REddyProc.php) [30]. Daily means of CO_2_ flux rates were computed with the gap-filled half-hourly NEE, ER, and GEP. Mean daily CO_2_ flux rates (μmol CO_2_ m^−2^ s^−1^, n = 48 d^−1^) were used to calculate the total C flux per day (g C m^−2^ d^−1^). The daily C balance was represented as: GEP = ER + NEP.

### 2.4 Aboveground biomass and leaf area measurements

Ten random sample sites were used for biomass measurements in 2018 and 2021 as previously described [3]. Leaves were separated from stems to measure single-sided leaf area with a leaf area meter (model 3100, LI-COR Inc.; Lincoln, NE). Grain yield at physiological maturity was achieved by hand clipping AGB from a 0.61 m segment of a soybean row segment spaced at 0.19 m (0.12 m^2^ in 2018) and 0.76 m (0.46 m^2^ in 2021).

### 2.5 Phenology characterization

On days when AGB samples were collected, we also recorded growth stages as described in a soybean field guide [31]. Additionally, canopy greenness was tracked with a webcam (model NetCam SC, StarDot Technologies). Daily data on canopy greenness, calculated as green chromatic coordinate – 90th percentile (GCC_90), were downloaded for the PhenoCam website, namely, mandanh5 (https://phenocam.nau.edu/webcam/sites/mandanh5/) and mandani2 (https://phenocam.nau.edu/webcam/sites/mandani2/). Retrieval, post-processing of the timeseries, and calculation of the phenological transition dates was accomplished using phenocamR R package [32]. Phenophase transition dates, based GCC, were found using a spline interpolation method when 5 % and 100 % of the GCC_90 amplitude on the raising phase to define the start of the green up and peak GGC phases. The start of senescence transition date was defined at 80 % of the GCC_90 amplitude on the falling phase for H5_2018_, I2_2018_, and H5_2021_. The start of senescence for I2 in 2021 was manually adjusted to DOY 248 because 80 % of the GCC 90 amplitude occurred during a transitory decline in GCC, The standardized GCC_90 was calculated by:

(1) $$GC{C\_90}_{standarized}=Smoothe{d\_90}_{Gcc\;day\;i}-Smoothe{d\_90}_{Gcc\;on\;planting\;date}$$

The end of senescence transition date was defined as the day when GCC_standardized_ was less than zero on the falling phase.

### 2.6 Agronomic and biomass data analysis

Harvest index was determined by dividing grain yield by AGB_max._ Water use efficiency based on yield (WUE_g_) was determined by dividing grain yield by total evapotranspiration (ET). Agronomic and biomass attribute differences between fields were evaluated using a pairwise *t*-test implemented using the Holm adjustment using the rstatix R package [33].

### 2.7 Big leaf estimation of meteorological, physical, and physiological attributes

The big leaf estimates were calculated using the functions in the Big Leaf R package [26,34] following the tutorial on big leaf (https://cran.r-project.org/web/packages/bigleaf/vignettes/bigleaf_tutorial.pdf) using the half-hourly data from the eddy covariance towers. The data were filtered for GCC maturity phenophase, PPFD greater than 200 μmol m^−2^ s^−1^, friction velocity (ustar) greater than 0.2 m s^−1^, gap filled VPD (VPD_f) greater than 0.1 hPa, gap filled latent heat flux (LE_f) greater than 0 W m^−2^, data quality flags (H_qc, LE_qc, NEE_fqc, Tair_fqc, and VPD_fqc) less than 1, canopy conductance to water vapor (g_sw_) between 0 and 1 mol m^−2^ s^−1^, and total accumulation precipitation less than 0.2 mm within a 24 h rolling window. Leaf dimension (Dl) was set as 0.05 m. Daily leaf area index (LAI) and canopy height (zr) for the big leaf calculations were estimated from a nonlinear least squares regression using a self-starting logistic model of the LAI and height timeseries from the periodic field sampling of LAI and plant height. Aerodynamic conductance (G_ah_) employed the boundary layer resistance formulation from Thom [35]. Roughness parameters were approximated using the roughness.parameters function with the “wind_profile” method and the fraction of displacement height on canopy height (frad_d) set as 0.7. Surface conditions were estimated with the surface.conditions function using the saturation vapor pressure formula by Allen [36]. The decoupling coefficient (omega) was calculated using the saturation vapor pressure formula [36] and the approach by Martin [37]. All other parameters not available in the half-hourly data were set to default in the Big Leaf R package.

## 3 Results

### 3.1 Agronomic performance

**Fig. 1:**
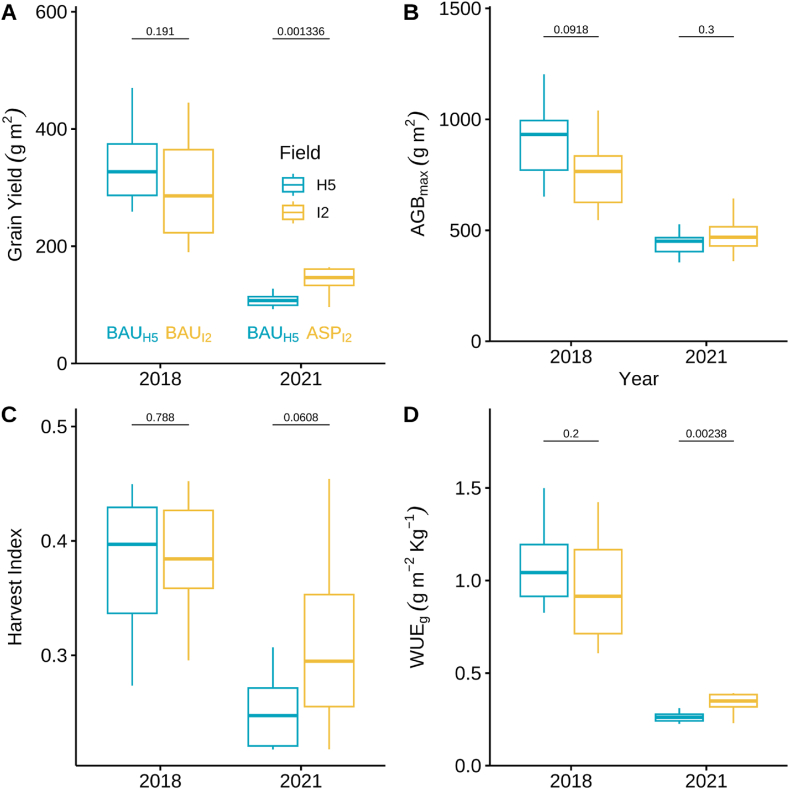
Agronomic attributes of soybean grown under business-*as*-usual and aspirational management strategies in 2018 and 2021. Boxplots of yield (A), maximum above ground biomass (AGB_max_, B), harvest index (C), and water-use efficiency based on yield (WUE_g_, D) at 10 sampling locations in each field. Light blue (BAU_H5_) and yellow (BAU_I2_ or ASP_I2_) represent the two fields managed under the business-*as*-usual (BAU) or aspirational management strategy (ASP). Numbers above the horizontal lines represent the adjusted p-values from a pairwise *t*-test implemented using the Holm adjustment. (For interpretation of the references to colour in this figure legend, the reader is referred to the Web version of this article.)

The two fields had lower yields, maximum above ground biomass (AGB_max_), harvest index (HI) and water-use efficiency based on grain yield (WUE_g_) in 2021 compared to 2018 due to the drought in 2021 (Fig. 1A, C, 1D). However, in 2021 ASP_I2_ performed better than the BAU_H5_ in terms of yield (mean_BAU(H5)_ ± se = 108 ± 4 and mean_ASP(I2)_ ± se = 143 ± 7 g m^−2^, *P* = 0.001; Fig. 1A), and WUE_g_ (mean_BAU(H5)_ ± se = 0.31 ± 0.001 and mean_ASP(I2)_ ± se = 0.42 ± 0.02; *P* = 0.0026, Fig. 1D). Maximum above ground biomass and harvest index were similar in both fields for both years (Fig. 1B and C), but ASP_I2_ had greater HI variability compared to BAU_H5_ in 2021.

### 3.2 Phenology

**Fig. 2:**
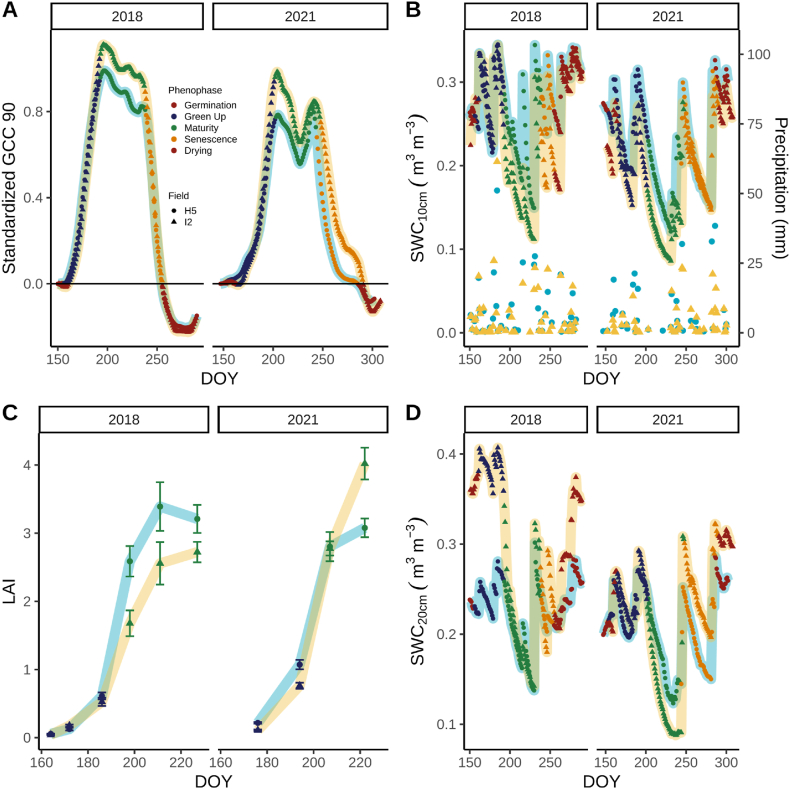
Soybean above-ground growth phenology and soil-water content in 2018 and 2021. Time series of smoothed green chromatic coordinate (G_cc_90_, A), soil-water content at 10 cm (SWC_10cm_, B), daily precipitation (B), leaf area index (LAI, C), and soil-water content at 20 cm (SWC_20cm_, D). Red, blue, green, and orange points represent beginning/end of season, green up, maturity, and senescence phenophases, respectively. Light blue circles, font, or outline (BAU_H5_) and yellow triangles, font, or outline (BAU_I2_ or ASP_I2_) represent the field managed under the business-*as*-usual (BAU) or aspirational (ASP) management strategy. (For interpretation of the references to colour in this figure legend, the reader is referred to the Web version of this article.)

To compare the photosynthetic phenology of these two fields, images from a Phenocam. The green-up and maturity phenophases derived from GCC corresponded to broadly classed vegetative and reproductive growth stages (Table 2). The start of green-up (SOG), when the G_cc_90_ exceeded 5 % of the G_cc_90_ amplitude, occurred earlier in BAU_H5_ (DOY_2018_ = 156; DOY_2021_ = 155) than the BAU_I2_ (DOY_2018_ = 161) or ASP_I2_ (DOY_2021_ = 166; Fig. 2A and Fig. S1; Table 2). However, the duration of the green-up phenophase was shorter in BAU_I2_ (32 days) and ASP_I2_ (36 days) compared to BAU_H5_ (37 and 49 days, 2018 and 2021, respectively; Table 2). G_cc_90_ was lower during maturity in BAU_H5_ compared to BAU_I2_ or ASP_I2_ in 2018 and 2021 (Fig. 2A). The start of senescence (SOS) began on DOY 238 the BAU_H5_ and BAU_I2_ in 2018. The GCC maturity phenophase was 5 days shorter in BAU_H5_ field compared to the ASP_I2_ in 2021, but the green up phenophase was 13 days longer in the BAU_H5_ (Table 1). Standard growth stage timing was similar in both fields in 2018 and 2021.

**Table 2:** Phenological characterization of soybean under contrasting years and management treatments. Duration (n), first day of the phenophase (start), growth stages observed for each phenophase, sums of daily precipitation (PCPN), evapotranspiration (ET), net ecosystem production (NEP = −NEE), gross ecosystem production (GEP), and ecosystem respiration (ER) and averages of underlying water-use efficiency (uWUE) during the 2018 and 2021 growing season in two fields (H5 and I2) managed under the business-*as*-usual (BAU) or aspirational management strategy (ASP). Totals or averages are reported for the green-up, maturity, and senescence phenophases and entire growing season.

Field/Year	Phenophase	Duration	Start	Growth Stages	PCPN	ET	GEP	ER	NEP	uWUE
		Days	DOY	Stage (DOY)	mm		g C m^−2^			g C kPa^0.5^ kg^−1^ H_2_O
BAUH5 -2018	Green Up	37	156	VC(163) – R1(186)	149	90	136	133	3	2.64 ± 0.21
	Maturity	45	193	R2(198) – R5(227)	107	150	407	237	171	3.49 ± 0.12
	Senescence	17	238	–	30	39	65	81	−15	1.91 ± 0.11
Total or Average		99	–	–	286	279	608	451	159	2.95 ± 0.11
BAUI2 -2018	Green Up	32	161	VC(164) – R1(186)	148	85	131	113	18	2.79 ± 0.16
	Maturity	45	193	R2(198) – R5(227)	89	147	369	199	171	3.31 ± 0.08
	Senescence	18	238	–	36	35	56	72	−15	2.05 ± 0.10
Total or Average		95	–	–	273	267	556	384	173	2.93 ± 0.09
BAUH5 -2021	Green Up	49	155	VE(162) – V9(194)	108	175	111	146	−35	1.57 ± 0.10
	Maturity	41	204	R1(207) – R5 (222)	37	132	140	119	21	1.98 ± 0.10
	Senescence	41	245	–	80	71	29	86	−56	1.24 ± 0.10
Total or Average		131	–	–	225	378	280	351	−70	1.60 ± 0.06
ASP_I2_ – 2021	Green Up	36	166	VE(162) – V9(194)	33	114	85	106	−21	1.86 ± 0.16
	Maturity	46	202	R1(207) – R5(222)	81	166	216	161	55	2.20 ± 0.11
	Senescence	43	248	–	83	88	52	102	−50	1.38 ± 0.08
Total or Average		125	–	–	197	368	353	369	−16	1.84 ± 0.08

In 2018, LAI was slightly greater during the GCC maturity phenophase in BAU_H5_ (mean ± se = 3.2 ± 0.2; DOY = 227) compared to BAU_I2_ (mean ± se = 2.7 ± 0.2; DOY = 227; Fig. 2C). Despite the dry conditions during 2021, LAI remained relatively high in BAU_H5_ (mean ± se = 3.1 ± 0.1; DOY = 222) but was greater in ASP_I2_ (mean ± se = 4.0 ± 0.2; DOY = 222; Fig. 2C).

Soil-water content fluctuations paralleled precipitation events (Fig. 2B and D). In 2021, precipitation was scarcer during the first half of the GCC maturity phenophase (Fig. 2B), when soil-water content at 10 cm depth (SWC_10__cm_) declined to a low of 0.12 and 0.09 m^3^ m^−3^ on DOY 231 in BAU_H5_ and ASP_I2_, respectively. Much needed and more frequent precipitation at that time caused SWC_5cm_ to increase to 0.30 m^3^ m^−3^ (DOY 246) in BAU_H5_ and 0.26 m^3^ m^−3^ (DOY 248) in ASP_I2_ by the start of senescence. The timing of these precipitation events coincided with the SOS transition date (Fig. 2A and B; Table 2). The ASP_I2_ field received 44 mm more precipitation during the GCC maturity phenophase.

In 2018, SWC_20cm_ started the growing season greater in BAU_I2_ than BAU_H5_, but during most of the GCC maturity phenophase both fields had similar SWC at this depth (Fig. 2D). In 2021, differences in SWC_20cm_ between the two fields were more notable during the GCC maturity and senescence phenophases. Soil-water content at 20 cm depth declined to 0.12 m^3^ m^−3^ (DOY 234) in BAU_H5_ but decreased more in ASP_I2_ (0.09 m^3^ m^−3^; DOY 237) during the GCC maturity phenophase, indicating that soybean roots in ASP_I2_ were better able to extract water from the soil (Fig. 2D). Interestingly, SWC_20cm_ increased more in the ASP_I2_ (0.30 m^3^ m^−3^ DOY 248; 233 %Δ) than BAU_H5_ (0.25 m^3^ m^−3^; DOY 245; 108%Δ) indicating greater water infiltration in ASP_I2_.

### 3.3 Carbon dioxide and water fluxes

**Fig. 3:**
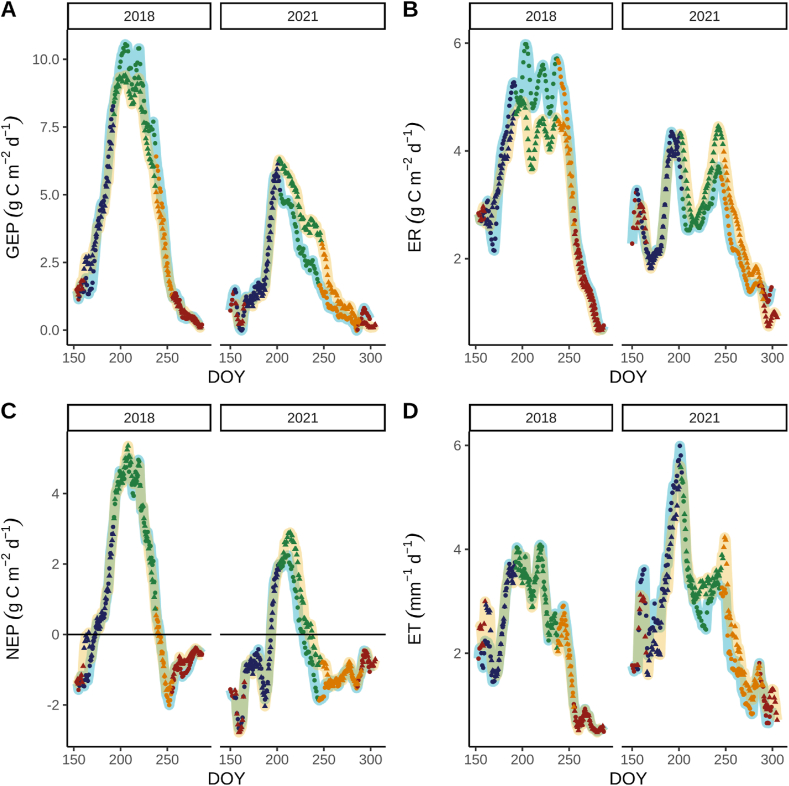
Moving averages of daily carbon dioxide fluxes and evapotranspiration rates during the phenophases of soybean grown under business-*as*-usual management in 2018 and business-*as*-usual and aspirational management in 2021 every 7 days. Times series of gross-ecosystem production (GEP, A) ecosystem respiration (ER, B), net ecosystem production (NEP, C), and evapotranspiration (ET, D) during the green up, maturity, and senescence phenophases. Light blue (BAU_H5_) and yellow (BAU_I2_ or ASP_I2_) represent the two fields managed under the business-*as*-usual (BAU) or aspirational management strategy (ASP). Red, blue, green, and orange points represent beginning/end of season, green up, maturity, and senescence phenophases, respectively. (For interpretation of the references to colour in this figure legend, the reader is referred to the Web version of this article.)

Daily gross ecosystem production (GEP), ecosystem respiration (ER), and net ecosystem production (NEP) were lower in 2021 compared to 2018 in both fields during GCC green up and maturity phenophases (Fig. 3A, B, and C; Table 2). However, evapotranspiration (ET) was greater in 2021 compared to 2018 (Fig. 3D; Table 2).

Field-specific differences in carbon dioxide and water fluxes were detected in 2021 during the green-up phenophase. During green up, total evapotranspiration was 42 % greater in BAU_H5_ compared to ASP_I2_, but total GEP was only 26 % greater in BAU_H5_ (Table 2). Carbon loss due to respiration was also 32 % greater in BAU_H5_ than ASP_I2_ during the GCC green up phenophase, resulting in much less NEP (Table 2).

Gross ecosystem productivity (GEP) was greater in ASP_I2_ (total GEP = 216 g C m^−2^) than BAU_H5_ (total GEP = 140 g C m^−2^; Fig. 3A) during GCC maturity phenophase, which contributed to 83 % more NEP in BAU_H5_ (Table 2). Coinciding with greater water uptake during GCC maturity (Fig. 2D), ASP_I2_ had 22 % more ET compared to BAU_I2_ during GCC maturity (Table 2). Therefore, uWUE was greater in ASPI_2_ (mean ± se = 2.20 ± 0.11 g C kPa^0.5^ kg^−1^ H2O) compared to BAU_H5_ (mean ± se = 1.98 ± 0.1 g C kPa^0.5^ kg^−1^ H_2_O; Table 2).

Across all GCC phenophases, total NEP in 2018 was similar in BAU_H5_ (159 g C m^−2^) and BAU_I2_ (173 g C m^2;^ Fig. 3C and D; Table 1). However, total NEP was greater in ASP_I2_ (−16 g C m^−2^) than in BAU_H5_ (−70 g C m^2^) in 2021.

### 3.4 Physiological ecosystem characteristics

**Fig. 4:**
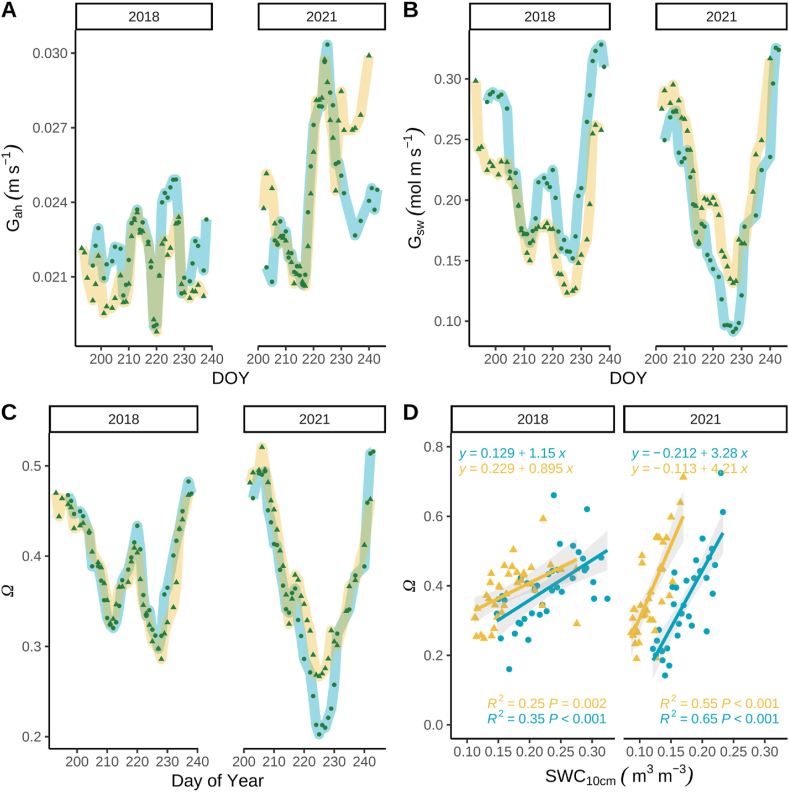
Seven day moving average daily time series of mean daytime aerodynamic conductance to heat transfer (G_ah_, A), surface conductance of water vapor (G_sw_, B), decoupling coefficient (Ω), and scatterplot and regression analysis of daily Ω and daily soil-water content at 10 cm (SWC_10cm_, D) of soybean grown under business-as usual and aspirational management strategies in 2018 and 2021 during the maturity phenophase. Light blue or circles (BAU_H5_) and yellow or triangles (BAU_I2_ in 2018 or ASP_I2_ in 2021) represent the two fields managed under the business-*as*-usual (BAU) or aspirational (ASP) management strategy. (For interpretation of the references to colour in this figure legend, the reader is referred to the Web version of this article.)

Analysis was restricted to the GCC maturity phenophase because an LAI greater than 2 is important for robust big-leaf parameterization when photosynthesis is most active. The calculation of aerodynamic conductance for heat transfer (G_ah_) and canopy conductance to water vapor (G_sw_) in the ‘big-leaf’ model permits the inference of physiological attributes. The average daytime G_ah_ was slightly greater in 2021 (BAU_H5_ = 0.025 ± 0.001 m s^−1^; ASP_I2_ = 0.025 ± 0.001 m s^−1^) than in 2018 (BAU_H5_ = 0.022 ± 0.001 m s^−1^; BAU_I2_ = 0.021 ± 0.001 m s^−1^; Fig. 4A).

**Fig. 5:**
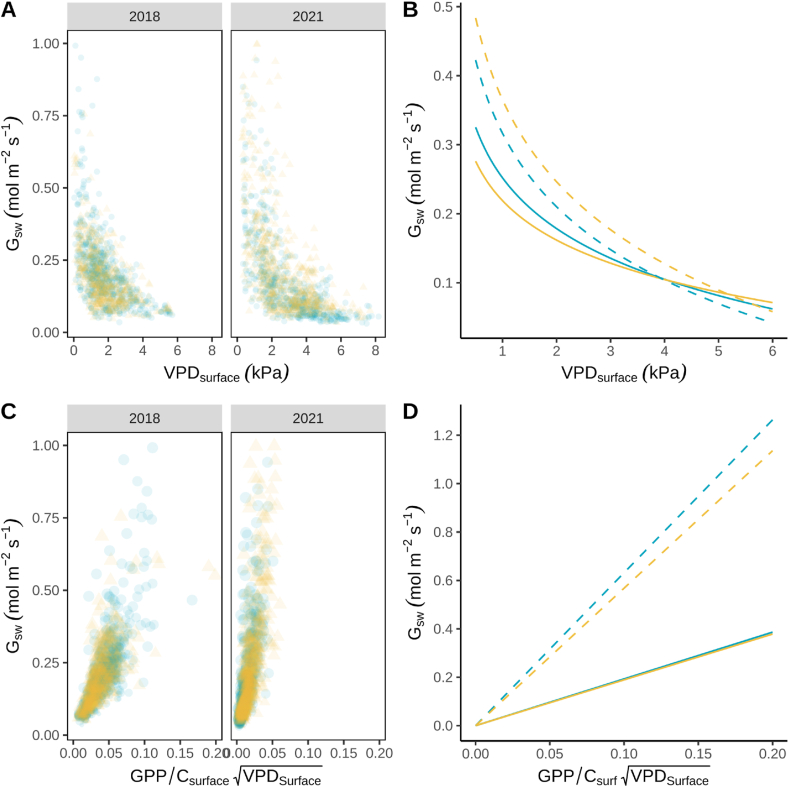
The relationship between surface conductance (G_sw_, A, B, C, and D) and surface vapor pressure deficit (VPD_surface_^,^ A and B) and gross-primary production adjusted for VPD and surface CO_2_ concentration (GPP/C_surface_(VPD_surface_^−0.5^), C and D) during the maturity phenophase. Light blue or circles (BAU_H5_) and yellow or triangles (BAU_I2_ or ASP_I2_) represent the two fields managed under the business-*as*-usual (BAU) or aspirational (ASP) management strategy. Solid lines represent model predictions from 2018 and dotted lines represent model predictions from 2021. (For interpretation of the references to colour in this figure legend, the reader is referred to the Web version of this article.)

Average daytime surface conductance of water vapor tended to be greater at the beginning and end of the GCC maturity phenophase (Fig. 4B). In 2018, G_sw_ was similar in BAU_H5_ (mean ± se = 0.20 ± 0.01 mol m^−2^ s^−1^) and BAU_I2_ (mean ± se = 0.19 ± 0.01 mol m^−2^ s^−1^, Fig. 5B). Daytime G_sw_ in 2021 were similar in both treatments (mean_ASPI2_ ± se = 0.21 ± 0.02 mol m^−2^ s^−1^ and mean_BAUH5_ ± se = 0.19 ± 0.02 mol m^−2^ s^−1^). However, daytime G_sw_ in 2021 was notably greater in ASP_I2_ (mean ± se = 0.15 ± 0.01 mol m^−2^ s^−1^) compared to BAU_H5_ (mean ± se = 0.10 ± 0.01 mol m^−2^ s^−1^) between DOY 220 and 232 when the SWC was low (Fig. 2, Fig. 4D).

The aerodynamic decoupling coefficient (omega, Ω), an indicator of the physiological regulation of ET, can be calculated from G_ah_ and G_sw_ [26]. A high decoupling coefficient indicates that the field's canopy surface does not sense the environment (uncoupled), thus resulting in lower physiological regulation of ET with higher Ω. Omega followed the same pattern as G_sw_, with higher values at the beginning and end of the GCC maturity phenophase (Fig. 2C). In 2021, Omega was lower in BAU_H5_ (mean ± se = 0.22 ± 0.02) than ASP_I2_ (mean ± se = 0.39 ± 0.02) from DOY 220 to DOY 232.

To test this further, we plotted the relationship between omega and SWC_10cm_ and performed a regression analysis. Omega and SWC_10cm_ were positively associated, indicating that as the soil dried, ET became more coupled to physiological regulation through stomatal closure (Fig. 4D). The strength of this relationship was weaker in 2018 (R^2^ = 0.25 and 0.35, BAU_H5_ and BAU_I2_, respectively) than in 2021 (R^2^ = 0.65 and 0.55, BAU_H5_ and ASP_I2_, respectively; Fig. 4D). In 2021, soybean plants in BAU_H5_ had greater physiological control over ET at higher SWC_10cm_ (Ω=0.5 when SWC_10cm_ = 0.22 m^3^ m^−3^) than soybean plants in ASP_I2_ (Ω = 0.5 when SWC_10cm_ = 0.15 m^3^ m^−3^; Fig. 4D), indicating that plants in the BAU_H5_ field were more conservative at a higher SWC. However, the slope of this relationship was 25 % greater in ASP_I2_ compared to BAU_H5_, indicating ASP_I2_ was more sensitive to changes in SWC_10cm_ (Fig. 4D).

As VPD_surface_ increased, G_sw_ decreased both years in both fields (Fig. 5A). The sensitivity of G_sw_ to VPD_surface_ (m) and G_sw_ at VPD_surface_ of 1 kPa (G_sw_ref_) were calculated using bootstrap resampling of the half-hourly data. The estimated sensitivity of G_sw_ to VPD_surface_ (m) and G_sw_ref_ was greater in 2021 compared to 2018 (Table 3). In 2018, soybeans in BAU_H5_ were more sensitive to VPD_surface_ than soybeans in BAU_I2_, but in 2021, soybeans in ASP_I2_ were more sensitive to VPD_surface_ (Table 3). The G_sw_ at VPD_surface_ of 1 kPa was also greater in ASP_I2_ compared to BAU_H5_ in 2021 (Table 3). Using these estimates, the empirical relationships between G_sw_ and VPD_surface_ are depicted in Fig. 5B.

**Table 3:** Mean daytime estimated stomatal sensitivity to vapor pressure deficit (m), surface conductance of water vapor at 1 kPa VPD (G_sw_ref_) and stomatal slope (G_1,uso_) ± standard error. Estimates were derived from bootstrap resampling of 75 % of the half-hourly data with 300 iterations.

Year	Field	m	G_sw_ref_	G_1,uso_	n
		mol m^−2^ s ln(kPa)^−1^	mol m^−2^ s^−1^	kPa^0.5^	half-hourly observations
2018	BAU_H5_	0.106 ± 0.001	0.252 ± 0.000	1.93 ± 0.004	586
2018	BAU_I2_	0.082 ± 0.000	0.219 ± 0.000	1.89 ± 0.004	534
2021	BAU_H5_	0.153 ± 0.001	0.316 ± 0.001	6.32 ± 0.014	567
2021	ASP_I2_	0.171 ± 0.001	0.365 ± 0.001	5.68 ± 0.012	613

**Fig. 6:**
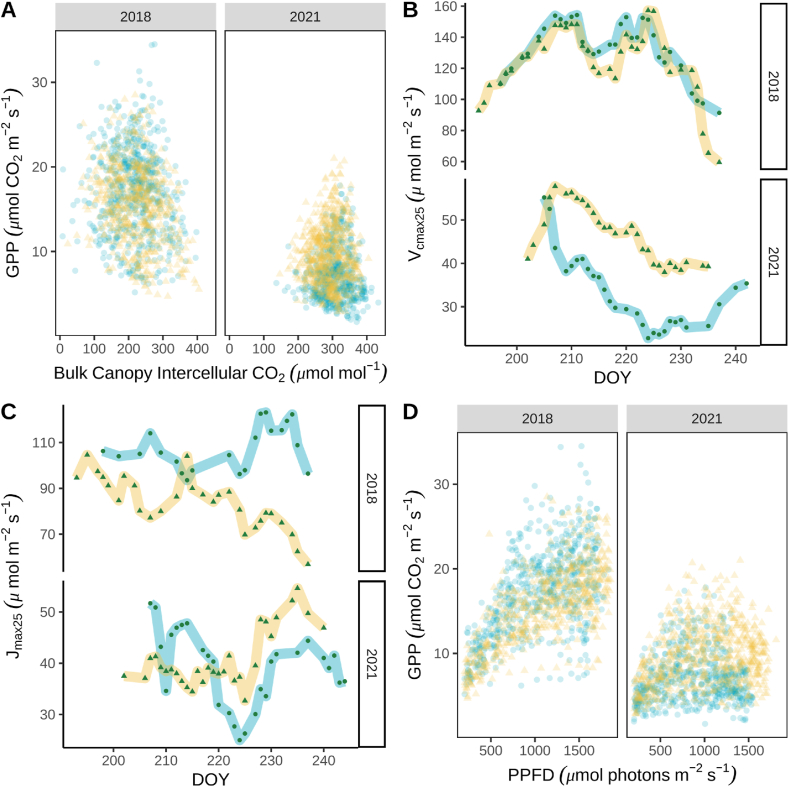
Photosynthetic capacity and light response during the maturity phenophase of soybean grown under business-*as*-usual and aspirational management strategies in 2018 and 2021. Gross primary productivity (GPP) response to bulk canopy CO_2_ response (A), seven-day moving average time series of estimated maximum carboxylation rate at 25 °C (V_cmax25_, B), maximum electron transport rate (J_max25_, C), and GPP response to photosynthetic photon flux density (PPFD, D). Light blue or circles (BAU_H5_) and yellow or triangles (BAU_I2_ or ASP_I2_) represent the two fields managed under the business-*as*-usual (BAU) or aspirational (ASP) management strategy. (For interpretation of the references to colour in this figure legend, the reader is referred to the Web version of this article.)

To characterize the intrinsic WUE (iWUE), the relationship between G_sw_ and gross primary productivity (GEP) adjusted for VPD and CO_2_ concentration was plotted (Fig. 6C). The slope of this relationship (G_1,uso_) provides an estimate for the stomatal slope parameter of the unified stomatal model [38]. A greater stomatal slope indicates a higher iWUE. The slope was greater in 2021 compared to 2018 (Fig. 5C), which agreed with the calculations of a lower WUE_g_ in 2018 (Fig. 1F). Furthermore, the GPP adjusted for VPD and CO_2_ was more limited in 2021 than in 2018, but the high G_sw_ occurred more frequently (Fig. 6C). The stomatal slope was estimated for each field using bootstrap resampling with the minimum surface conductance (G_o_) set to zero. The estimated stomatal slopes were greater in 2021 than in 2018 (Table 3; Fig. 5D). The estimated stomatal slope was 7.75 % greater in BAU_H5_ relative to BAUI_2_ in 2018, but in 2021 G_1,uso_ was 33 % greater in ASP_I2_ compared to BAU_H5_. Therefore, shifting to the aspirational management practice that included cover crops improve iWUE during the GCC maturity phenophase. This improvement in iWUE is consistent with the 27 % greater WUE_g_ (Fig. 1D) and 10 % greater uWUE (Table 2) in ASP_I2_ relative to BAU_H5_ in 2021.

Bulk canopy intercellular CO_2_ concentration was estimated using the calculated surface CO_2_ concentration, G_sw_ and GPP_._ The average daily daytime bulk intercellular CO_2_ in both fields was lower in 2018 (mean ± se = 219 ± 2 μmol mol^−1^) compared to 2021 (mean ± se = 298 ± 2 μmol mol^−1^). This suggested that dry conditions in 2021 lowered the photosynthetic capacity of soybean plants. Plotting the relationship between the half-hourly bulk canopy intercellular CO_2_ concentration and GPP revealed lower GPP at higher bulk intercellular CO_2_ concentrations (Fig. 6A), confirming the lower photosynthetic capacity in 2021. Moreover, the distribution of points in Fig. 6A indicated greater photosynthetic capacity in the BAU_H5_ in 2018 and the ASP_I2_ in 2021. The maximum carboxylation rate at 25 °C (V_cmax25_) and maximum electron transport rate (J_max25_) at the canopy level were calculated daily using the big-leaf framework. Both estimates of photosynthetic capacity were greater in 2018 compared to 2021, and field-specific differences were discernible (Fig. 6B and C). Average daily V_cmax25_ were the same in the BAU_H5_ (mean ± se = 130 ± 6 μmol m^−2^ s^−1^) and BAU_I2_ (mean ± se = 124 ± 8 μmol m^−2^ s^−1^) fields in 2018, but V_cmax25_ was 42 % greater in ASP_I2_ (mean ± se = 46 ± 2 μmol m^−2^ s^−1^) compared to BAU_H5_ in 2021 (mean ± se = 30 ± 2 μmol m^−2^ s^−1^; Table 4). Average daily J_max25_ was greater in BAU_H5_ (mean ± se = 106 ± 6 μmol m^−2^ s^−1^) than BAU_I2_ (mean ± se = 85 ± 6 μmol m^−2^ s^−1^) in 2018, but J_max25_ was 13 % greater in ASP_I2_ (mean ± se = 42 ± 3 μmol m^−2^ s^−1^) than BAU_H5_ (mean ± se = 37 ± 3 μmol m^−2^ s^−1^; Table 4) in 2021.

**Table 4:** Daytime estimated photosynthetic capacity and light-response curve parameters. Values represent the mean ± standard error. Maximum carboxylation rate at 25 °C (V_cmax25_) and maximum electron transport rate (J_max25_) estimates represent the photosynthetic capacity, α represents the ecosystem quantum yield, and GPP_ref_ represents the gross primary productivity at 2000 μmol m^−2^ s^−1^ photosynthetic flux density. The estimates were calculated daily and averaged across the maturity phenophase. Light blue or circles (BAU_H5_) and yellow or triangles (BAU_I2_ or ASP_I2_) represent the two fields managed under the business-*as*-usual (BAU) or aspirational (ASP) management strategy.

Year	Field	V_cmax25_	J_max_	α	GPP_ref_
		μmol m^−2^ s^−1^	μmol m^−2^ s^−1^	μmol m^−2^ s^−1^/μmol m^−2^ s^−1^	μmol m^−2^ s^−1^
2018	BAU_H5_	130 ± 6	106 ± 6	0.0547 ± 0.0002	21.17 ± 0.02
2018	BAU_I2_	124 ± 8	85 ± 6	0.0415 ± 0.0003	19.59 ± 0.03
2021	BAU_H5_	30 ± 2	37 ± 3	0.0808 ± 0.0009	7.79 ± 0.01
2021	ASP_I2_	46 ± 2	42 ± 3	0.0544 ± 0.0003	10.96 ± 0.01

Plotting the relationship between GPP and PPFD using half-hourly data indicated differences in the light-response curves between the two fields (Fig. 6D; Table 4). Canopy photosynthesis at saturating light intensity appeared to be greatest in BAU_H5_ in 2018 but lowest in BAU_H5_ in 2021 (Fig. 6D). Estimates of CO_2_ assimilation at light saturation (GPP_reff_) and light-use efficiency (α) were modeled using a rectangular hyperbolic light response curve with bootstrap resampling. Estimated GPP_ref_ was only 7.8 % more in the BAU_H5_ (mean ± se = 21.17 ± 0.02 μmol m^−2^ s^−1^) than BAU_I2_ (mean ± se = 19.59 ± 0.03 μmol m^−2^ s^−1^) in 2018, but in 2021 the GPP_ref_ was 33 % more in ASP_I2_ (mean ± se = 10.96 ± 0.01 μmol m^−2^ s^−1^) than BAU_H5_ (mean ± se = 7.79 ± 0.01 μmol m^−2^ s^−1^).

## 4 Discussion

The yield performance of soybean grown in fields managed under business-*as*-usual and aspirational management practices diverged during a multi-year drought. The aspirational system that included cover crops and residue retention produced 29 % greater soybean yields than the field managed without cover crops (Fig. 1B). It is difficult to attribute this performance difference to initial differences between the two fields because of the similar agronomic performance and soil characteristics of the two fields in 2016 (Table S1 and Fig. 1B). Two factors driving the divergent performance in 2021 can be identified by considering the soil-water content at 20 cm depth (Fig. 2D). First, SWC_20cm_ decreased more during GCC maturity phenophase in the ASP_I2_; this indicates soybean roots or associated microorganisms in the ASP_I2_ were able to access and extract more water and nutrients from this depth compared to the conditions in the BAU_H5_. Secondly, SWC_20cm_ rebounded more in the ASP_I2_ due to precipitation events that occurred between August 19th (DOY 231) and September 3rd (DOY 246) at the end of the GCC maturation phenophase and beginning of senescence (Fig. 2C and D), indicating that the aspirational management practices may have improved water infiltration. However, soil-structural changes have yet to be detected through ongoing measurements. Alternatively, residue from overwintering cover crops from the corn phase may be providing additional soil cover during the soybean phase, which could lower evapotranspiration rates immediately following precipitation events. Since aboveground biomass accumulation was similar in both fields, but GEP was greater in ASP_I2_, we hypothesize more photosynthate was being allocated to root growth in ASP_I2_.

Another related driver of the performance divergence between the ASP_I2_ and BAU_H5_ in 2021 was the timing of GCC phenological transitions. Green-up occurred more quickly in the ASP_I2_ than the BAU_H5_ (Table 2), which resulted in less total evapotranspiration in the ASP_I2_ than the BAU_H5_ early in the growing season (Table 2). The faster green up and improved water uptake in the ASP_I2_ facilitated a longer GCC maturity phenophase (Fig. 2A–Table 2) and greater water use (Table 2) during the GCC maturity phenophase. Similar to our results, Phenocam based-monitoring of winter wheat phenology indicates management practices can have profound effects on the timing of GCC-based phenology with robust effects on crop yield [33].

The aerodynamic decoupling between the surface and the atmosphere, Ω, followed the same pattern as G_sw_ (Fig. 4B and C). Aerodynamic decoupling and G_sw_ were initially high at the beginning and end of the GCC maturity phenophase (Fig. 4B and C). The decoupling coefficient is a gauge of the relative dependence of canopy transpiration on physical or stomatal properties. When Ω approaches zero the sensitivity of transpiration to changes in stomatal conductance and VPD is greater. As SWC_10cm_ lost moisture, Ω decreased in 2018 and 2021, but the slopes of these relationships were greater in 2021 (Fig. 4D). Omega in the ASP_I2_ was more sensitive to changes in SWC_10cm_ compared to the BAU_H5_ in 2021, and the Ω -SWC_10cm_ relationship was shifted to the left in the ASP_I2_. This indicates that soybeans in the ASP_I2_ were better able to tolerate dry soil conditions, and a lower SWC_10cm_ was required to promote the physiological regulation of evapotranspiration. Root signals that promote stomatal closure probably required drier soil conditions in the ASP_I2_ compared to the BAU_H5_. Alternatively, the root hydraulic system in ASP_I2_ may have been able to extract more soil water than BAU_H5_ during drought in 2021. Therefore, the relationships between SWC, stomatal conductance, and transpiration can be modified by soil and root properties that are influenced by agricultural management practices. The similar AGB in BAU_H5_ and ASP_I2_ despite GEP differences in 2021 suggest that soybean in ASP_I2_ had greater root biomass and denser root surface area, which are important traits for drought tolerant soybean [39].

The response of G_sw_ to VPD_surface_ followed an exponential decline in 2018 and 2021 (Fig. 5A and B). The shape of this relationship is different than a previous leaf-level study with soybean that reported a linear relationship between VPD_surface_ and stomatal conductance (g_w_) in California [40]. Under drought conditions, G_sw_ tended to be greater under low VPD_surface_ and less under high VPD_surface_ (Fig. 5A and B; Table 3). Furthermore, the G_sw_-VPD_surface_ curve was shifted upward in ASP_I2_ compared to BAU_H5_ in 2021. Therefore, G_sw_ tended to be greater in ASP_I2_ relative to the BAU_I2_ regardless of the VPD_surface_. By keeping G_sw_ relatively high, soybean in ASP_I2_ may have mitigated some of the effects of thermal stress by reducing CO_2_ starvation and thereby decreasing light stress. Drought conditions caused G_1,uso_ to increase in 2021 in both fields, but G_1_,uso was greater in ASP_I2_ compared to BAU_H5_ fields in 2021.

Dry conditions in 2021 resulted in lower photosynthetic capacity relative to 2018. However, the photosynthetic capacity was greater in ASP_I2_ compared to BAU_H5_ in 2021. The improved photosynthetic capacity translated to improved photosynthesis rates when C_i_ was lower because of lower stomatal conductance. Therefore, CO_2_ starvation was less acute in the ASP_I2_ than the BAU_H5_ during the GCC maturity phenophase. The improved photosynthetic capacity in the ASP_I2_ in 2021 can be attributed to a consistently greater V_cmax25_ and frequently greater J_cmax_. Previous research indicates that elevated temperatures, as they were in 2021, can have positive, negative, or neutral effects on V_cmax25_ and J_max25_ depending on the duration, intensity, and soybean growth stage [[41], [42], [43]]. A possible explanation for the improved photosynthetic capacity of soybean in the ASP_I2_ is a positive feedback loop with improved photosynthesis resulting more energy available for nitrogen fixation.

The finding that management approach may influence V_cmax_ and J_max25_ under drought conditions suggests agricultural management practices can improve soybean photosynthetic rates. Soybean breeding efforts have increased yields by improving light interception, light-use efficiency, and partitioning biomass into seed production [44], but breeding have had a limited impact on soybean photosynthetic capacity [9]. Koester et al. (2016) hypothesized that soybean breeding efforts have focused on developing cultivars adapted for moist soil conditions. Our research suggests that one strategy that may improve soybean productivity under dry conditions is to use winter cover crops to facilitate more aggressive water uptake by soybean.

Net ecosystem production (NEP) is only positive during the cash-crop growing season in the NGP under the prevailing management practice in non-drought conditions. Previous research indicates that reduced tillage and the inclusion of cover crops can meaningfully improve soybean NEP [45]. Our results indicate that the aspirational management practice helped preserve soil carbon by maintaining a positive NEP during the 2021 drought, whereas the business-*as*-usual practice resulted in a negative NEP (Table 2). This improvement can be attributed to more photosynthesis (GEP) rather than less ecosystem respiration (Table 2). If the NEP is consistently lower in fields managed using the business-*as*-usual practices, then we would expect divergence in soil carbon between the fields over time. This divergence could create a positive feedback loop whereby the benefits of the aspirational treatment could become more substantial over time.

The results presented here are consistent with previous work indicating conservation agriculture can improve soybean yield stability [19,[46], [47], [48], [49]] and can increase soybean yields by improving soil attributes [50]. However, cover crops have been reported to decrease yields in subsequent soybean crops during drought conditions in Brookings, SD [49]. Therefore, our results and previous results indicate that inclusion of cover crops can have inconsistent effects on soybean performance in the NGP. The source of this inconsistency is unclear, but it could be caused to greater springtime soil water use by the cover crop in South Dakota compared to North Dakota where overwinter cover crop survival may be lower and the springtime growing season is shorter. Soybean yield have previously been reported to be negatively impacted by residue retention in Ontario [14,15], but these studies did not include cover crops or drought. Additional research is needed to understand the factors responsible for these inconsistent effects. It is likely that the timing, duration, and intensity of precipitation events, as well as soil temperature and nutrient availability, explain some of this variability.

We acknowledge that this study involves tradeoffs between the ability to make statistical inferences and research costs. Eddy covariance flux towers and the relatively large land area they require are expensive, therefore replication is unrealistically cost prohibitive. However, biomass sampling at 10 sites in each field provides an indication of the spatial variation. It is also not possible to untangle cause and effect due to the specific strategies used in the aspirational treatment. For example, care must be taken when attributing performance differences to either cover crops or residue retention specifically, but this study was designed to compare cropping systems of our local producers. This study provides valuable information about how alternate management systems already being implemented by producers in the NGP can affect crop physiology and ecosystem provisioning. The temporal resolution of this study provides the basis for testable hypotheses that can be addressed through future pot, plot, and field level experiments. An ongoing plot-scale experiment will allow us to test these hypotheses in the future with greater statistical rigor.

This study provides mechanistic insight into how aspirational management strategies can improve soybean drought resiliency in the NGP by targeting soil properties that can facilitate water and nutrient uptake by soybean roots. The application of the ‘big leaf’ framework suggests this approach could be a useful tool for making standardized ecosystem diagnostic comparisons of business-*as*-usual and aspirational management practices across the wider LTAR network. LTAR sites, like the NGP site, that are growing crops at their geographic fringe can be important for understanding field-scale stress physiology. Outcomes can be used to model regional and global consequences of cropland management practices and guide future research about the interactions between genetics and management.

## 5 Conclusions

Cover crops and residue retention, as components of a management system in the NGP, improved soybean performance under drought conditions. Soybean in the aspirational field had better water-use efficiency during the GCC green up and maturity phenophases and were better able to extract soil water during the GCC maturity phenophase. These differences allowed the soybean in the aspirational field to have greater canopy conductance regardless of the vapor pressure deficit, greater photosynthetic capacity, greater photosynthetic rates, greater LAI, and a longer GCC maturity phenophase. These differences functioned together resulting in an increased harvest index and grain yields. Consequently, this study indicates that management practices that includes residue retention and cover crops can be beneficial for soybean under drought conditions by improving water uptake.

## Data availability

Data and R code used in this research are publicly available on Zenodo (https://zenodo.org/records/8335085) after September 30, 2024.

## CRediT authorship contribution statement

**Craig W. Whippo:** Writing – review & editing, Writing – original draft, Visualization, Validation, Formal analysis, Data curation. **Nicanor Z. Saliendra:** Writing – review & editing, Writing – original draft, Project administration, Methodology, Investigation, Formal analysis, Data curation. **Mark A. Liebig:** Writing – review & editing, Writing – original draft, Supervision, Project administration, Methodology, Investigation, Funding acquisition, Conceptualization.

## Declaration of competing interest

The authors declare that they have no known competing financial interests or personal relationships that could have appeared to influence the work reported in this paper.
