# Supplementary material for: Cover crop inclusion and residue retention improves soybean production and physiology in drought conditions
Source: Heliyon. 2024 Apr 20;10(8):e29838. doi: 10.1016/j.heliyon.2024.e29838 (PMC11063448; doi:10.1016/j.heliyon.2024.e29838)

SUPPLEMENTAL MATERIALS

Table S.1. Baseline soil conditions at 0-5, 5-10, and 10-30 cm in spring 2016 for fields H5 and I2 at the LTAR Northern Plains Croplands Common Experiment research site, Mandan, ND, USA.

	**- - - - - 0-5 cm - - - - -**		**- - - - - 5-10 cm - - - - -**		**- - - - - 10-30 cm - - - - -**	
**Variable (units)**	**Field H5**	**Field I2**	**Field H5**	**Field I2**	**Field H5**	**Field I2**
Soil bulk density (Mg m^{-3})	1.12 (0.01)*	1.23 (0.02)	1.43 (0.02)	1.48 (0.03)	1.29 (0.01)	1.32 (0.02)
Electrical conductivity (dS m^{-1})	0.34 (0.01)	0.28 (0.02)	0.24 (0.01)	0.22 (0.01)	0.23 (0.01)	0.24 (0.03)
Soil pH (-log[H^+])	5.33 (0.09)	5.67 (0.10)	5.41 (0.06)	5.73 (0.07)	6.22 (0.05)	6.51 (0.13)
Soil organic matter (%)	4.91 (0.14)	4.50 (0.09)	3.69 (0.10)	3.34 (0.12)	2.90 (0.11)	2.65 (0.11)
Sand (g kg^{-1})	186 (8)	186 (13)	183 (10)	196 (11)	209 (23)	200 (13)
Silt (g kg^{-1})	553 (12)	539 (13)	515 (10)	510 (12)	482 (24)	493 (20)
Clay (g kg^{-1})	261 (7)	275 (4)	302 (7)	294 (4)	309 (7)	307 (11)

* Standard error shown in parentheses.

Figure S1. Phenocam images at phenology transition dates. The white number in the field images represent Julian day of the year (DOY) at the start of green up (SOG), start of maturity (SOM), start of senescence (SOS), and end of senescence (EOS).

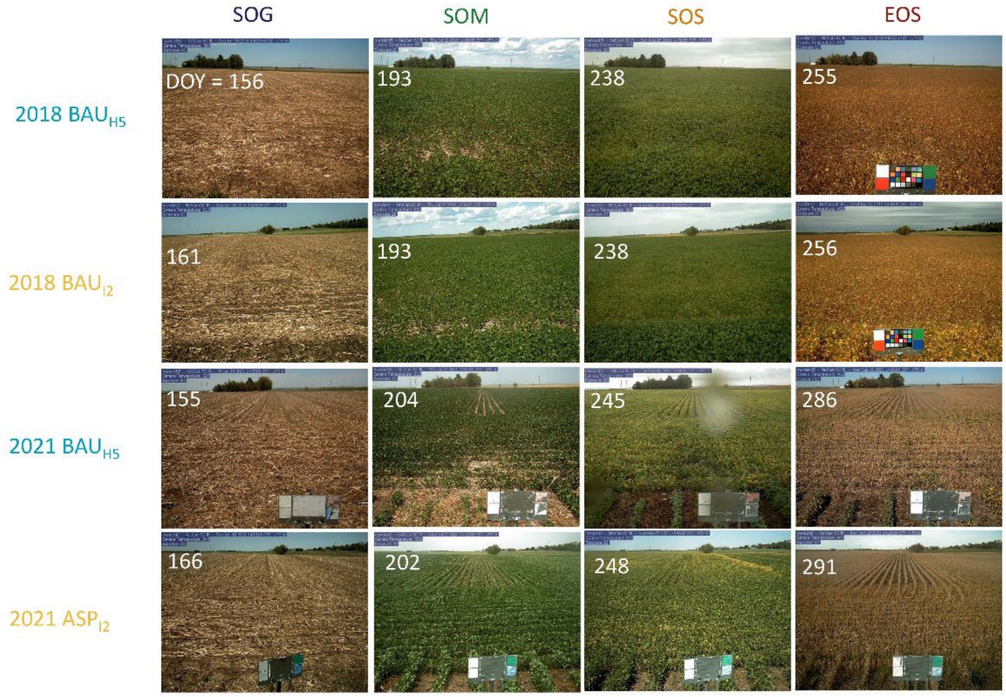

Supplement: Multimedia component 1 [file mmc1.pdf]
